# Real-Life Treatment Intervals and Morphological Outcomes Following the Switch to Faricimab Therapy in Neovascular Age-Related Macular Degeneration

**DOI:** 10.3390/jpm15050189

**Published:** 2025-05-06

**Authors:** Katrin Löw, Vasilena Sitnilska, Yuhe Tang, Jeany Q. Lammert, Tim U. Krohne, Lebriz Altay

**Affiliations:** Department of Ophthalmology, Faculty of Medicine and University Hospital of Cologne, University of Cologne, 50937 Cologne, Germany; vasilena.sitnilska@uk-koeln.de (V.S.); yuhe.tang@uk-koeln.de (Y.T.); jeany.lammert@uk-koeln.de (J.Q.L.); tim.krohne@uk-koeln.de (T.U.K.); lebriz.altay@uk-koeln.de (L.A.)

**Keywords:** neovascular age-related macular degeneration, anti-VEGF therapy, faricimab, intravitreal injection

## Abstract

**Objectives**: To evaluate the efficacy of faricimab in patients with neovascular age-related macular degeneration (nAMD) that did not respond to other VEGF inhibitors. **Methods**: This retrospective study included the eyes of patients diagnosed with nAMD who had been switched to faricimab treatment due to the persistence of intraretinal fluid (IRF) and/or subretinal fluid (SRF), despite monthly anti-VEGF treatment with aflibercept, bevacizumab, or ranibizumab using the treat and extend regimen, and who had received at least three faricimab injections following the switch. Best-corrected visual acuity (BCVA) measurement and optical coherence tomography (OCT) analysis were performed at each visit, and the OCT results were graded by two independent readers. **Results**: We included 41 eyes of 39 patients (21 male, 18 female) with a mean age of 80.5 ± 8.1 years. The median duration of anti-VEGF treatment prior to the switch to faricimab was 5.0 years, with a median of 53 injections. Complete resolution of IRF and SRF was observed after the first dose of faricimab in 12 eyes (29.3%) and after the third dose in 15 eyes (36.6%). Twenty-eight eyes reached a follow-up time after a switch of at least 12 months, with a median of 10 faricimab injections. Of these 28 eyes, 10 eyes (35.7%) exhibited complete IRF/SRF resolution; treatment intervals were extended beyond 4 weeks in 21 eyes (80.7%), and 8 eyes (28.6%) presented complete IRF/SRF resolution under extended treatment intervals at month 12. Central retinal thickness after 12 months was reduced from a median of 368.0 µm to 297.5 µm (*p* < 0.001), and the BCVA remained stable (*p* = 0.057). No adverse events were reported throughout the entire treatment period. **Conclusions**: In nAMD patients with poor anti-VEGF treatment response, complete and fast fluid resolution and the extension of treatment intervals can be reached by switching to faricimab, even after years of prior unsuccessful therapy.

## 1. Introduction

Age-related macular degeneration (AMD) is one of the leading causes of severe, irreversible vision loss in developed countries [1,2,3,4]. Considering the demographic changes of an aging population, the number of people affected will continue to increase [5]. Although the majority of patients are affected by non-neovascular AMD, its neovascular form is responsible for most cases of severe vision loss. The introduction of intravitreal injections of anti-vascular endothelial growth factor (VEGF) has revolutionized the therapeutic possibilities in the field of AMD and significantly improved its prognosis [6,7,8]. Nevertheless, a significant proportion of patients require monthly intravitreal anti-VEGF injections for years to stabilize the condition, which represents a burden for practitioners, patients, and health economics [9].

Faricimab is the first targeted antibody with a dual mechanism of action—binding both VEGF-A and angiopoetin-2—that has been approved for neovascular AMD (nAMD) patients. In the worldwide pivotal studies titled TENAYA and LUCERNE, the noninferiority of best-corrected visual acuity (BCVA) was shown even for treatment intervals of 16 weeks, in comparison to 8 weekly doses of aflibercept [10]. Little is known about the efficacy of faricimab in pretreated eyes with a poor treatment response and only a few studies have investigated the long-term outcomes (≥12 months) in these cases [11,12,13].

In this retrospective study, we aimed to evaluate the efficacy of faricimab in patients with nAMD who did not respond to other VEGF inhibitors, in both the short and long terms.

## 2. Materials and Methods

### 2.1. Study Design and Endpoints

This retrospective study included nAMD patients with a poor response to anti-VEGF treatment with aflibercept, bevacizumab, and ranibizumab when following the treat and extend regimen. All the included patients were switched to faricimab between October 2022 and April 2024. Poor response was defined as the persistence of any detectable intraretinal fluid (IRF) or subretinal fluid (SRF) despite monthly injections of VEGF inhibitors reviewing the last three treatment intervals before the switch. Only those patients who received a minimum of 3 faricimab injections following the switch were included. The data were analyzed in pseudonymized form.

The main endpoint was the interval extension (post-switch versus pre-switch). Secondary endpoints included BCVA to describe the functional outcome, as well as morphological criteria such as central retinal thickness (CRT), the presence of SRF, IRF, retinal pigment epithelium (RPE) atrophy, subfoveal disciform scars, reticular pseudodrusen, and outer retinal tubulations (ORTs).

Spectral-domain optical coherence tomography scans (SD-OCT; scan area 20° × 15° (5.8 × 4.4 mm), with 37 B-scans, and a distance between B-scans of 122 µm) were assessed at baseline (before the first faricimab injection) and after the first and third faricimab injections, as well as after 12 and 18 months of faricimab therapy, if available. Two image graders (L.A. and K.L.) independently reviewed the SD-OCT scans for the above-mentioned OCT parameters. In case of disagreement, a third grader (V.S.) reviewed the OCT images. Dry OCT scans were defined as the absence of SRF and IRF. Subfoveal disciform scars (subfoveal fibrosis) were defined as either sub-RPE or subretinal scars involving the central 500 µm of the SD-OCT scan [14]. Foveal (within the central 500 µm) and extrafoveal (outside of the central 500 µm) atrophy was defined as complete RPE and outer retinal atrophy (cRORA) with a zone of hypertransmission of ≥250 µm [15]. Central retinal thickness was measured from the internal limiting membrane to Bruch’s membrane (BrM). Outer retinal tubulations (ORTs) were defined as degenerating photoreceptors arranged in a circular or ovoid fashion, presenting as branching tubules [16].

The inclusion criteria were:

- Diagnosis of neovascular AMD;
- Age ≥ 60 years;
- Previous anti-VEGF treatment with at least one of the following agents:
- aflibercept, bevacizumab, ranibizumab;
- Poor response to anti-VEGF treatment, as defined above.
- The exclusion criteria were:
- Treatment-naïve patients;
- Patients who received fewer than 3 faricimab injections;
- Patients with other concomitant retinal/macular pathologies that could affect the results;
- Participation in a pivotal faricimab study.

### 2.2. Treatment Protocol

Intravitreal injections were administered following the adapted treat and extend (T&E) protocol of the University of Cologne, without mandatory loading doses (a modified early T&E regimen). Routine post-injection monitoring was performed according to local recommendations.

### 2.3. Statistical Analysis

Descriptive measures were calculated for demographic, clinical, and treatment variables. To describe normally distributed values, mean ± standard deviation (SD) values were used. Non-normally distributed values were presented as median and interquartile range (IQR) values. Absolute and relative frequencies were calculated for binary variables. To assess statistical differences between groups, Student’s *t*-test was used for normally distributed values, whereas for non-normal distribution, the Mann–Whitney-U test was applied. For binary variables, the chi^2^-test value was calculated. To assess changes in BCVA, CRT, and treatment intervals, the Wilcoxon signed-rank test for paired samples was applied. Since only a limited number of statistical tests were performed, no Bonferroni correction was applied. A *p*-value of ≤0.05 was considered statistically significant.

Statistical analyses were performed with SPSS version 29.0 (SPSS Inc., Chicago, IL, USA).

## 3. Results

### 3.1. Cohort Description

In this retrospective study, 41 eyes of 39 patients (21 male, 18 female) with a mean age of 80.5 ± 8.1 were analyzed. Baseline demographic data are shown in Table 1. Patients received a median of 53 (IQR 32–73) intravitreal anti-VEGF injections for the treatment of nAMD and were observed for a median of 5 (IQR 2–7) years before they were switched to faricimab. Before the switch, 25 eyes had been treated with three different VEGF inhibitors (aflibercept, bevacizumab, and ranibizumab).

In total, 28 eyes reached the long-term follow-up date of 12 months with a median of 10 (IQR 8–11) faricimab injections; 13 eyes were observed for a period of 18 months with a median of 15 (IQR 11–17) injections. Of the remaining patients, 13 did not reach 12 months of follow-up for several reasons: 9 patients had a too-short observation time after the switch, 1 patient passed away, 1 was switched to another substance due to a deterioration in OCT under faricimab, 1 did not continue with the injections for health reasons, and, for 1 patient, treatment was stopped because of foveal scarring.

Subfoveal disciform scars were present in 24 of the 41 studied eyes (58.5%) at baseline, of which 21 were sub-RPE scars and 3 were mixed-type sub-RPE and subretinal scars. cRORA in the fovea was present in 9 eyes (22%) at baseline, whereas 15 eyes (36.6%) presented extrafoveal cRORA. Five eyes (12.2%) presented ORTs at baseline, while four eyes (9.8%) presented reticular pseudodrusen. None of the eyes presented subretinal hemorrhages. Table 2 displays their OCT characteristics at baseline.

### 3.2. Functional Outcome

Compared to baseline, BCVA changed significantly after the first faricimab injection, with a median (IQR) of 0.4 (0.3–0.6) at baseline and 0.4 (0.2–0.55) after one injection (*p* = 0.04). Looking at the visual acuity after three injections, there was no statistically significant change in BCVA compared to baseline (0.4 vs. 0.4; *p* = 0.544). In the subgroups of eyes with follow-ups of 12 and 18 months, there was also no significant change in BCVA (M12: 0.35 vs. 0.35; *p* = 0.057; M18: 0.3 vs. 0.2; *p* = 0.051).

### 3.3. Morphological Outcome

**Figure 1:**
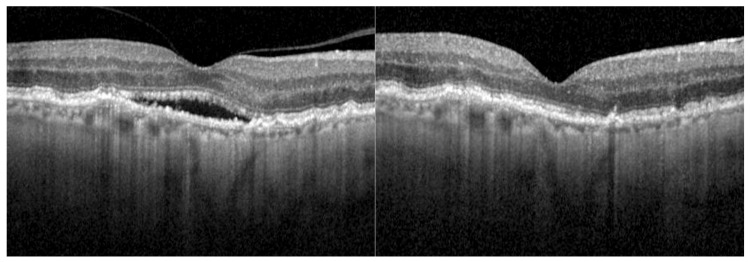
OCT scan of a right eye at the baseline examination and after 12 months of faricimab treatment. This eye had been diagnosed with neovascular age-related macular degeneration 3 years prior to the treatment switch and had been treated with 42 injections of other anti-VEGF inhibitors.

**Figure 2:**
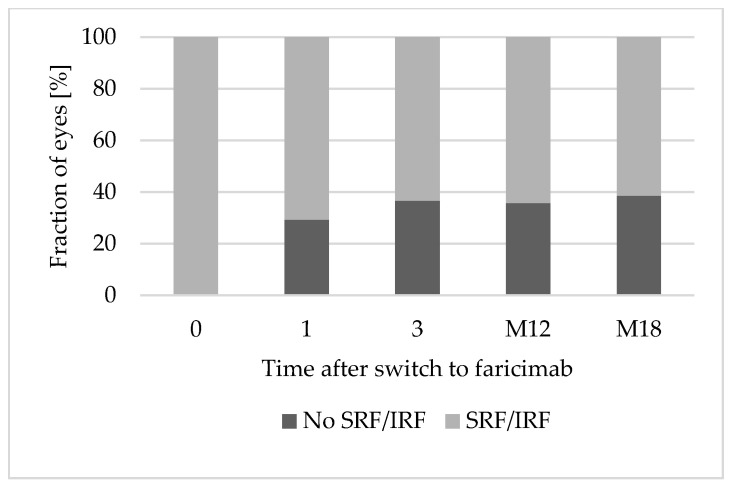
Complete resolution of SRF and IRF over 18 months. Abbreviations: 0: baseline; 1: after 1 faricimab injection; 3: after 3 faricimab injections; M12: after 12 months; M18: after 18 months. IRF: Intraretinal fluid. SRF: Subretinal fluid.

At baseline, all eyes presented retinal fluid in the OCT. After the first faricimab injection, 12 eyes (29.3%) achieved a completely dry OCT scan. After three faricimab injections, 15 of 41 eyes (36.6%) were completely dry. Of the 28 eyes reaching a follow-up of 12 months, 10 eyes (35.7%) showed a dry OCT after 1 year of treatment with faricimab. By month 18, the OCT was dry in 38.5% (5 of 13 eyes). Figure 1 shows OCT scans of an eye with nAMD at baseline and after 12 months of treatment with faricimab. Figure 2 shows the fraction (%) of eyes with complete resolution of SRF and IRF over 18 months.

CRT was significantly reduced after the first faricimab injection (365 µm vs. 301 µm; *p* < 0.001), while the number of eyes presenting foveal cRORA (n = 9) was stable compared to baseline.

**Figure 3:**
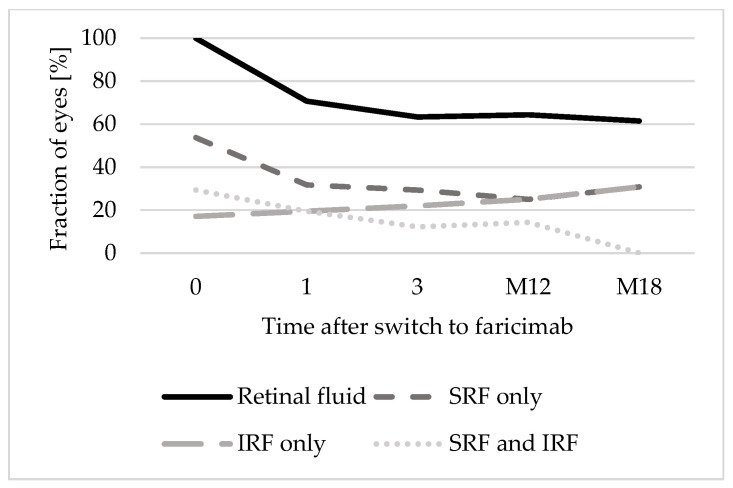
Distribution of retinal fluid types over 18 months. Abbreviations: 0: baseline; 1: after 1 faricimab injection; 3: after 3 faricimab injections; M12: after 12 months; M18: after 18 months. IRF: Intraretinal fluid. SRF: Subretinal fluid.

At baseline, 12 eyes (29.3%) presented both SRF and IRF, 22 eyes (53.7%) presented only SRF, and 7 eyes (17.1%) presented only IRF. The proportion of eyes presenting only SRF (31.7% after 1 faricimab injection; 30.8% after 18 months) and both SRF and IRF (19.5% after 1 faricimab injection; 0% after 18 months) decreased over time. However, the proportion of eyes presenting IRF in OCT increased (19.5% after 1 faricimab injection; 30.8% after 18 months). Figure 3 shows the distribution of types of retinal fluid (only SRF, only IRF, or both SRF and IRF) over 18 months.

At long-term follow-ups of 12 and 18 months, respectively, there was a significant reduction in CRT compared to baseline, (M12: 368 µm vs. 297.5 µm; *p* < 0.001, compared with M18: 433 µm vs. 348 µm; *p* < 0.001).

### 3.4. Treatment Interval Extension

Before the switch to faricimab, all 41 eyes were treated at a median of 4-weekly intervals and still presented retinal fluid.

**Figure 4:**
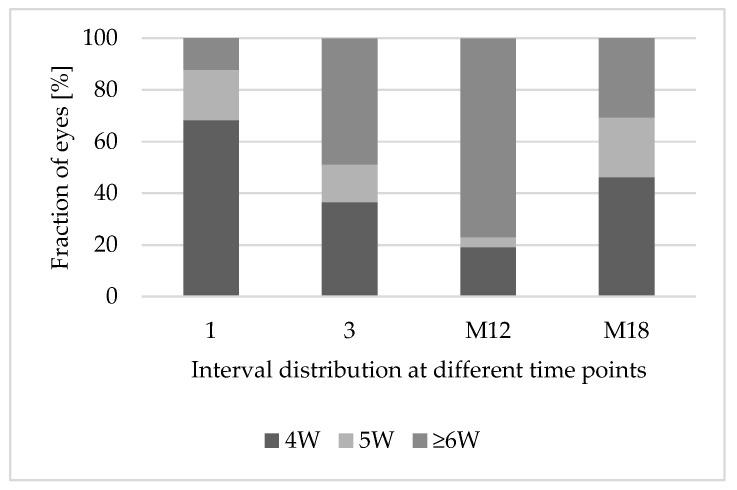
Treatment interval extension after switching to faricimab. Abbreviations: 1: after 1 faricimab injection; 3: after 3 faricimab injections; M12: after 12 months; M18: after 18 months; 4W: treatment interval of 4 weeks; 5W: treatment interval of 5 weeks; ≥6W: treatment interval of at least 6 weeks.

After the first faricimab injection, treatments of 12.2% (n = 5) of eyes could be extended to treatment intervals of ≥6 weeks. After the third faricimab injection, treatments of 48.8% (n = 20) were extended to treatment intervals of ≥6 weeks. In the subcohort of patients with eyes reaching 12 months of follow-up, treatment intervals could be extended significantly (6 weeks vs. 4 weeks; *p* < 0.001). Two eyes needed to be excluded because they were injected later than the intended time, due to adherence problems, while 76.9% (n = 20) of eyes reached intervals of ≥ 6 weeks. Figure 4 demonstrates the treatment interval distribution at different time points over 18 months.

### 3.5. Prognostic Factors

To determine the potential prognostic factors for reaching a dry OCT in the long term, we compared the characteristics of the fraction of patients with a dry OCT after 12 months and those with persisting retinal fluid. There was neither a statistically significant difference for demographic values such as age and sex, nor one for variables concerning previous anti-VEGF therapy (Table 3). For baseline OCT parameters, there was also no statistically significant difference in the groups.

### 3.6. Safety

There were no complications such as endophthalmitis or retinal vasculitis reported with faricimab treatment in this retrospective study. Two patients had a history of endophthalmitis in the years before switching to faricimab.

## 4. Discussion

The aim of this retrospective study was to evaluate the real-world efficacy of faricimab in a cohort of pre-treated nAMD patients not responding to VEGF inhibitors. This analysis shows that the extension of treatment intervals and morphological improvement while maintaining a stable function is possible in a cohort of eyes with a long history of previous anti-VEGF treatment.

Poor treatment response and frequent injections increase the treatment burden and adherence problems, especially in patients with a long history of anti-VEGF injections and partly irreversible OCT lesions, such as fibrotic scars and RPE atrophy. Therefore, the elongation of treatment intervals while still allowing disease control is of high research interest [14,17,18].

For treatment-naïve patients, functional and morphological improvement under faricimab therapy could be shown in the pivotal studies of TENAYA and LUCERNE. With its dual inhibition of angiopoetin-2 and VEGF-A, faricimab seems to allow an extension of treatment intervals with sustained efficacy compared to aflibercept and an even greater effect on reducing the biomarkers of vascular instability by controlling both neovascularization and vascular leakage [10,19]. However, little is known about the outcomes in a real-world setting of pre-treated nAMD cases.

Treatment switching is a common practice for addressing nonresponse in eyes that are being treated with VEGF inhibitors for nAMD [20]. Reasons for this poor response, such as tachyphylaxis along with decreased bioefficacy after repeated intravitreal injections with the same substance, are discussed elsewhere [21,22]. Due to the lack of recognized definitions of non- or suboptimal response, we administered strict inclusion criteria to minimize bias [23].

There was no significant long-term change in BCVA compared to baseline in our study. Only a slight improvement in visual acuity was observed after the first injection. In the literature, information on BCVA varies greatly. Some authors have reported improvement [12,24,25] or stability [23,26,27,28], but deterioration is also observed [29]. This may primarily be due to varying inclusion and exclusion criteria and varying durations of previous anti-VEGF therapy. In our study, we observed a cohort of patients with long histories of a median of 5 years of anti-VEGF injections, presenting partly irreversible OCT lesions at baseline. ORTs, as a sign of degenerating photoreceptors, were present in 12.2% of the eyes [16]. In total, 58.5% presented subfoveal disciform scars, while 22% presented RPE atrophy located in the fovea. According to the literature, these lesions correlate with reduced BCVA, confirming the limited potential for functional improvement in our cohort [14,17]. These morphological lesions, which were already present at baseline, could explain the discrepancy between the morphological and functional results.

Morphological improvement was shown even after long-term follow-ups of 12 and 18 months. Resolution of the retinal fluid could be observed in a high proportion of cases, even after 12 and 18 months (35.7% and 38.5%), and CRT was reduced significantly (*p* < 0.001). While the proportion of eyes with SRF was reduced over time, the proportion of eyes presenting only IRF slightly increased. Similar results were reported in other real-world studies, mostly those with shorter observation periods and varying sample sizes [23,24,25,26,27,28,29,30]. It is known that SRF responds better to anti-VEGF therapy and is associated with a lower rate of progression to cRORA than IRF [31,32]. The presence of IRF is associated with a poorer visual prognosis [33].

In general, a reduction in CRT is not only a consequence of fluid resolution but can also be caused by a development or progression of cRORA and thinning of the outer retina [15]. This may contribute to CRT reduction in our cohort and may also partly explain the limited functional improvement reported and the above-mentioned gap between functional and morphological improvement. However, the absolute number of eyes with foveal cRORA was stable during the observation period.

Most importantly, treatment intervals could be extended significantly in our cohort in terms of both short-term and long-term follow-ups. Notably, an interval extension with concomitant morphological improvement was observed in our cohort of patients exhibiting a nonresponse to other VEGF inhibitors (median of 5 years previous therapy, and median of 53 injections), unfavorable functional prognosis, and advanced age (80.5 ± 8.1 years). In short-term studies, interval extension or at least the maintenance of treatment intervals with stable OCTs could be observed as well [24,34,35]. For other follow-ups of 12 months, data are limited, but Rush and Cancian et al. reported a possible interval extension in patients who switched from ranibizumab or aflibercept to faricimab [12,13]. The available data, as well as our study, suggest that a switch to faricimab may reduce the treatment burden while allowing good disease control, even with different therapy regimens. While Cancian et al. administered a loading of four faricimab injections every 4 weeks, Rush et al. administered three 4-weekly faricimab injections [12,13]. Patients in our study were treated following a modified, early T&E regimen, without mandatory upload. Considering the fact that nAMD is a chronic disease causing a high treatment burden for patients as well as for practitioners that is challenging for healthcare systems, therapies with lower injection frequencies but offering safe and effective disease control are of great importance [9].

Still, we would like to state that after 5 years of previous continuous treatment, a significant proportion of patients require very frequent injections to maintain already impaired visual acuity. Compared to pivotal studies of faricimab and long-term studies with other VEGF inhibitors, the therapy results and the reduction of the treatment burden in our pre-treated, complex patient sample are reduced [10,17]. In addition, the morphological improvement and interval extension are not necessarily attributable to the substitution of faricimab alone, but may also partly be explained by the treatment switch per se [36].

Regarding safety, we did not observe any adverse events such as endophthalmitis or retinal vasculitis throughout the entire observational period when performing routine post-injection monitoring. Although faricimab has been proven to be comparable to aflibercept in terms of safety, there have still been cases of intraocular inflammation or retinal vasculitis reported in the literature [10,37,38,39]. Our rather small sample size may underestimate the potential side effects of intravitreal therapy with faricimab.

The questions of patient selection and the ideal time point for a successful switch to faricimab were not addressed in this study. Statistically significant prognostic factors could not be identified. In the literature, OCT and other markers have been discussed, but there are still no recognized markers predicting therapy response in nAMD patients [40,41]. For now, there are no clear recommendations and guidelines regarding the exact time and criteria for when to switch from another substance to faricimab. Prospective studies with larger cohorts will be necessary to address these questions.

Limitations of this study include its retrospective, single-center design with a relatively small sample size and the potential for selection bias. These aspects may lead to the limited generalizability of the results.

## 5. Conclusions

In nAMD patients with poor treatment responses, a high number of cases reached a dry OCT status under faricimab, even after years of previous anti-VEGF treatment with other substances. The functional outcomes were limited, most likely due to chronic and partly irreversible foveal lesions such as RPE atrophy and subfoveal disciform scarring, but these could be maintained. Notably, an extension of the injection intervals was possible. With regard to safety, no unfavorable events were observed. The question of the ideal time point for a treatment switch remains unanswered. No prognostic factors for a successful treatment switch could be identified.

## Figures and Tables

**Table 1:** Demographic and baseline characteristics.

Demographic and Baseline Characteristic	
Number of eyes	41
Number of patients	39
Age in years (mean ± SD)	80.5 ± 8.1
Gender (male/female)	21/18
Laterality (left/right)	21/20
Lens status (pseudophakic/phakic)	32/9
BCVA (logMAR) (median; IQR)	0.4 (0.3–0.6)
Injections before switch (median; IQR)	53.0 (32.0–73.0)
Last substance administered Aflibercept/Bevacizumab/Ranibizumab	13/13/15
Total of different substances administered before switch 1/2/3	6/10/25

Abbreviations: BCVA: best-corrected visual acuity.

**Table 2:** OCT (Optical coherence tomography) baseline characteristics.

OCT Characteristic	N (%)
Reticular drusen	4 (9.8)
Retinal fluid	41 (100)
SRF and IRF	12 (29.3)
SRF only	22 (53.7)
IRF only	7 (17.1)
ORTs	5 (12.2)
Subfoveal disciform scar	24 (58.5)
Sub-RPE	21
Subretinal	0
Mixed sub-RPE and subretinal	3
cRORA foveal/extrafoveal	9/15 (22.0/36.6)
Subretinal hemorrhage	0
Central retinal thickness (µm) (median; IQR)	365.0 (292.5–453)

Abbreviations: cRORA: complete outer retinal and RPE (retinal pigment epithelium) atrophy. IRF: Intraretinal fluid. ORT: Outer retinal tubulations. SRF: Subretinal fluid.

**Table 3:** Evaluation of prognostic factors.

	SRF/IRF M12 (N = 18)	No SRF/IRF M12 (N = 10)	*p*-Value
Age (mean ± SD)	81.22 ± 8.03	79.6 ± 7.52	0.605
Sex (female)	8	6	0.622
BCVA (logMAR) (median; IQR)	0.3 (0.2–0.53)	0.45 (0.3–0.5)	0.555
Years until switch (median; IQR)	6.5 (2–7.25)	6.5 (2.75–8.0)	0.944
Injections until switch (median; IQR)	64.5 (30.25–81.0)	51.5 (31.5–61.5)	0.245
CRT at baseline (µm) (median; IQR)	396.25 (320.5–585.25)	333.5 (276.5–400.38)	0.051
Presence of reticular drusen at baseline	2	2	0.520
Presence of SRF at baseline	14	9	0.418
Presence of IRF at baseline	10	3	0.194
Presence of ORTs at baseline	2	1	0.927
Presence of fibrosis at baseline	13	4	0.094
Presence of foveal cRORA at baseline	2	3	0.211
Presence of extrafoveal cRORA at baseline	6	3	0.856

Abbreviations: BCVA: best-corrected visual acuity. cRORA: Complete RPE and outer retinal atrophy. CRT: Central retinal thickness. IRF: Intraretinal fluid. ORT: Outer retinal tubulation. SRF: Subretinal fluid.

## Data Availability

The dataset is available on request from the authors.

## Abbreviations

The following abbreviations are used in this manuscript:

(n)AMD	(neovascular) age-related macular degeneration
BCVA	best-corrected visual acuity
BrM	Bruch’s membrane
cRORA	complete RPE and outer retinal atrophy
CRT	central retinal thickness
ILM	internal limiting membrane
IQR	interquartile range
IRF	intraretinal fluid
ORT	outer retinal tubulations
RPE	retinal pigment epithelium
SD	standard deviation
SD-OCT	spectral domain optical coherence tomography
SRF	subretinal fluid
T&E	treat and extend regimen
VEGF	vascular endothelial growth factor
